# Modeling Adhesive Anchors in a Discrete Element Framework

**DOI:** 10.3390/ma10080917

**Published:** 2017-08-08

**Authors:** Marco Marcon, Jan Vorel, Krešimir Ninčević, Roman Wan-Wendner

**Affiliations:** 1 Christian Doppler Laboratory LiCRoFast, Department of Civil Engineering and Natural Hazards, University of Natural Resources and Life Sciences (BOKU), 1190 Vienna, Austria; mmarco88@gmail.com (M.M.); jan.vorel@fsv.cvut.cz (J.V.); nincevick@gmail.com (K.N.); 2 Department of Mechanics, Faculty of Civil Engineering, Czech Technical University in Prague, 16629 Prague, Czech Republic

**Keywords:** bonded anchors, discrete elements, fastenings, bond-slip law, combined failure, photogrammetry

## Abstract

In recent years, post-installed anchors are widely used to connect structural members and to fix appliances to load-bearing elements. A bonded anchor typically denotes a threaded bar placed into a borehole filled with adhesive mortar. The high complexity of the problem, owing to the multiple materials and failure mechanisms involved, requires a numerical support for the experimental investigation. A reliable model able to reproduce a system’s short-term behavior is needed before the development of a more complex framework for the subsequent investigation of the lifetime of fasteners subjected to various deterioration processes can commence. The focus of this contribution is the development and validation of such a model for bonded anchors under pure tension load. Compression, modulus, fracture and splitting tests are performed on standard concrete specimens. These serve for the calibration and validation of the concrete constitutive model. The behavior of the adhesive mortar layer is modeled with a stress-slip law, calibrated on a set of confined pull-out tests. The model validation is performed on tests with different configurations comparing load-displacement curves, crack patterns and concrete cone shapes. A model sensitivity analysis and the evaluation of the bond stress and slippage along the anchor complete the study.

## 1. Introduction

Post-installed mechanical and adhesive anchors provide the opportunity to design and build flexible new structures, but also to renovate or strengthen old buildings. The usage of fastening systems has been increasing steadily during the last few decades. In particular, bonded anchors are a valuable and relatively cost-effective fastening type with a different working principle compared to more traditional mechanical anchors. Mechanical anchors typically transfer load to concrete at the anchor’s lower end due to mechanical interlocking and/or friction. For bonded anchors, the load is transferred through the thin adhesive mortar layer along the entire bonded length. The different working principle introduces other failure mechanisms that are not present for mechanical anchors, as they are related to (partial) bond failure (see [1]).

**Figure 1:**
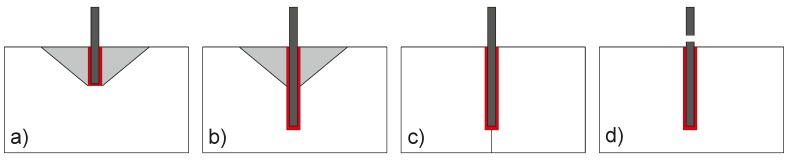
Possible failure mechanisms of bonded anchors: (**a**) concrete cone failure; (**b**) combined failure; (**c**) splitting failure; and (**d**) steel failure.

Figure 1 shows the typical failure mechanisms for bonded anchors under tension loading. The typical concrete cone failure (Figure 1a), which is the dominant scenario for anchors that operate by mechanical interlocking, is still possible for bonded anchors, but only for small embedment depths and high bond strengths. Further failure modes include concrete splitting and steel failure (Figure 1c,d, respectively). The combined failure (Figure 1b) is a more complex failure mechanism, which depends on the mechanical properties of concrete, on the embedment depth and on the bond strength.

Different ways have been pursued in the literature to predict the performance of adhesive adhesive anchors. They can be classified into (a) empirical (analytical) models calibrated on databases and (b) numerical models. Among the analytical models proposed for the combined concrete cone-bond failure mechanism, summarized by Cook et al. in [2], the simplistic uniform bond model showed excellent capability on pull-out capacity prediction. Furthermore, artificial neural networks, after training on a part of a world-wide experimental database, have been successfully used for the prediction of mechanical anchors’ (see [3,4]) and bonded anchors’ (see [5]) capacity.

With increasing computational resources and steadily improving modeling concepts, numerical modeling has become a reliable tool for structural analysis, but also for the investigation of local details such as fastening systems. Many concrete models have been developed during the last few years to describe the behavior of concrete, both for design and research purposes. Finite element models and discrete models approach the problem from different perspectives, and each one of them has advantages and disadvantages. Software used by engineers for structural analysis often does not contain accurate enough constitutive models for the base material as shown in [6], where natural stone is assumed, but the statement can be extended to concrete and masonry.

Finite element models based on the damage plasticity theory such as the CC3DNonLinCementitious2implemented in ATENA (see [7]), have a fracture-plastic constitutive model, which is a combination of fracturing behavior for tensile stresses and plastic behavior for compressive stresses. Required inputs are directly the concrete properties such as elastic modulus, compressive strength, tensile strength, fracture energy and some other parameters, which define the compressive and fracture behavior. This concrete constitutive model has been used successfully also for bonded anchor simulations (see [8]) comparing also 2D axisymmetric element and 3D element approaches.

Other classes of finite element constitutive models are based on the microplane theory (see, e.g., [9,10,11,12]) developed by Bažant. In these models, the constitutive relation is defined in terms of micro-stress and micro-strain vectors acting on planes of all possible orientations. This way, the definition of a constitutive law is conceptually simpler because it deals with vectors instead of tensors. Since the microplanes are randomly oriented, the oriented nature of cracking and slip can be captured easily. The drawback of this model is that it has many parameters that lack a clear physical meaning, as they describe the failure envelope on microplanes. Ožbolt et al. performed in the last few years many numerical simulations on anchors and anchor groups (see [13,14]) using their version of the microplane model. The concrete properties are directly used in the concrete constitutive model, and the bond strength is calibrated on confined tests. Once the model parameters are calibrated, different geometries and boundary conditions are used to validate the models’ capabilities. After the successful model validation, numerical studies can be performed in order to investigate cases that cannot be tested in the laboratory due to limitations in size, testing duration or number of required tests.

Other contributions can be found in the literature regarding bonded anchors’ modeling using a broad variety of concepts and based on material constitutive laws, e.g., [15,16].

Discrete models such as the lattice discrete particle model (LDPM) are a valuable alternative for concrete modeling. The LDPM developed by Cusatis and co-workers (see [17]) is a discrete concrete model that has been proven to be able to reproduce the behavior of concrete under a variety of different loading conditions (see [18]), once properly calibrated and validated. The LDPM mimics the heterogeneity of the concrete by randomly placing virtual aggregates in the domain defined by the specimen geometry following the sieve curve and considering the mix design. The random placement starts from a position determined by a seed number; thus, changing the seed number, the placement will change. Due to this fact, the model can also partially reproduce some of the variability seen in tests owing to the different internal structure of the specimens. As for experiments, also these numerical simulations need to be repeated a certain number of times to identify more accurately the mean response. Simulations of bonded anchors utilising discrete elements for concrete do not appear in the literature, to the author’s knowledge. The LDPM, can bring advantages with respect to the more traditionally-used finite element method (FEM) for anchors’ modeling because this model features unbiased fracture propagation without the need to impose a crack-band due to its inherent characteristic length and discrete nature. It, thus, predicts realistically any fracture process and provides realistic crack patterns. These features are very important especially in cases where the system response is determined by the interaction of multiple damage mechanisms or propagating cracks, i.e., two developing concrete break-out cones in an anchor group and or distributed damage due to material deterioration. The mimicked heterogeneity allows the localization of damage in the area of the largest incompatible strains and, hence, stresses. In the case of deterioration processes, the same feature ensures the development of damage as a result of local volumetric expansion, e.g., due to thermal expansion, creep and hygral shrinkage [19], or the gel formation as a result of the alkali-silica-reaction [20]. The development of a predictive life-time model for bonded anchors, also accounting for the evolution of material properties due to ongoing hydration (see [21]), benefits from these general features of LDPM. Furthermore, the heterogeneous discrete mesh following the particle distribution also leads to more realistic, generally not symmetric, stress and strain distributions associated with realistic cone shapes, as can be seen in Figure 10b. Consequently, symmetry cannot be exploited (as done frequently for FEM analysis; see, e.g., [14]), leading to comparably larger computational cost. Similar behavior can only be achieved in FEM analysis by introducing spatially-variable properties, e.g., based on random fields, as proposed by Pukl et al. [22]. These however are still topics of ongoing research and not yet clearly understood. Overall, the presented bonded anchor model in a discrete element framework is not intended to be used for day to day design. The model can be used as the basis for the derivation of more efficient yet safe future design codes and approval documents that are based on a rigorously-developed and verified scientific basis. In order to decrease the large computational cost of the discrete element simulations compared to standard finite element analysis, mass scaling has been used (the mass of the smallest LDPM elements is increased to ensure the stability of the numerical solution). Mass scaling has been limited to a maximum of 30% of the total specimen mass in order to keep dynamic effects on the system response at a negligible level.

The approach followed by this research team is to model the complete behavior of the singular components. Concrete and adhesive mortar are tested in order to determine their respective mechanical properties, depending on their curing degree, which is affected by time and the history of environmental conditions. Different geometries and curing conditions were tested for different ages of concrete. Compressive and tensile strength, elastic modulus and fracture energy were determined at 3, 8 and 28 days. Additionally, creep and shrinkage tests were performed to gain insights into the long-term behavior of concrete under sustained loads. Dog-bone specimens and compact tension specimens are tested for different curing levels to obtain elastic and fracture properties of the adhesive mortar. Furthermore, accelerated creep tests were performed to obtain the multi-decade creep behavior of this particular material. In order to transfer all of the material information to the system response, numerical simulations are required. The ultimate goal is to create a tool that is able to predict reliably the life-span of bonded anchor systems under sustained load, also in the presence of deterioration processes of various types, which can involve all or only single system components, i.e., the mortar layer, steel bar or the concrete member. However, a reliable long-term prediction model needs to be able to accurately predict the short-term behavior first. This publication focuses on the calibration and validation of a numerical model for bonded anchors under quasi-static tensile loading. For the first time, a discrete concrete model is used to systematically study the behavior of bonded anchors due to its realistic prediction of damage localization and propagation. Most likely, this is the source of the achieved excellent prediction quality, both in terms of load displacement response and failure mechanism. The experimental data used for this paper are created internally ensuring consistency and quality of the results and, thus, provide a reliable basis for the model development and validation.

## 2. Modeling Bonded Anchors at Different Scales

**Figure 2:**
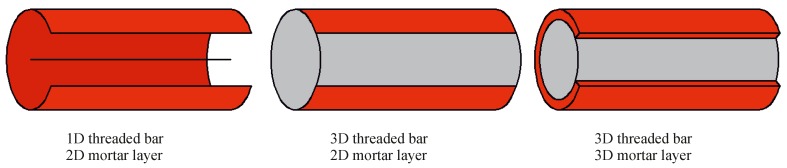
Different scales at which an anchor can be modeled.

Fastening systems can be represented, as any other structural system, at different scales, according to the scope of the investigation. In order to design a building, for instance, concrete is taken as a continuum elastic material. For local problems, such as moisture transport and concrete hydration in structural details on the other end, the cement paste and aggregates should be modeled, and the concrete should no longer be considered a continuum. A bonded anchor pull-out problem could be modeled with 2D axisymmetric elements or 3D elements. With the help of 2D axisymmetry, as for other 2D approximations such as plane stress or plane strain, a 3D problem can be reduced to a 2D problem. The necessary, but also sufficient conditions to simulate a real 3D problem via an axisymmetric 2D reduction are the symmetry of loads, geometry and material. Depending on the level of detail that one wants to meet, all three aspects of the problem or none of them can be considered axisymmetric. The intent of this paper is to propose a model that can be used later on e.g. for sustained load simulations, which involve the aging of the components, heat transfer and moisture diffusion, concrete pre-damage and different loading conditions. The hypothesis of perfect symmetry would be ideal for the specific case of pure tension, but too restrictive or even prohibitive for more general future applications. Derived from the above-mentioned considerations, a 3D model for the concrete base material seems to be required to deal with fastening system problems. Regarding the anchor and the mortar layer, as can be seen in Figure 2, three configurations are of interest: the anchor itself can be modeled as a 1D beam or discretized in 3D by finite elements, while the mortar layer can be represented as a 2D interface or, again, as a three-dimensional body.

Generally, the best choice is the simplest model that satisfies a given set of constraints, and since, at the moment, the interaction with the steel failure and micro-cracking of the adhesive mortar layer are not of interest, the modeling concept based on a 1D threaded bar and a 2D mortar interface have been selected.

### 2.1. Threaded Bar

One of the assumptions of the experimental campaign is that in all of the pull-out tests, the threaded bar remains in its linear elastic regime. For this specific reason, threaded bars of steel class 12.9 have been used for all of the pull-out tests. To simulate the anchor, a simple linear elastic material and 1D beam elements (the formulation of which is based on the Bernoulli–Euler beam theory) have been used. However, for systems where the local stress in the steel elements reaches higher levels than the yield strength, a more realistic elasto-plastic behavior should be used instead of the simpler elastic relationship. The choice of the proper constitutive law, as shown in [23], is essential to be able to model properly the system behavior.

### 2.2. Lattice Discrete Particle Model

The LDPM is a concrete model that simulates the mechanical interaction of coarse aggregates embedded in a cementitious matrix. The full description of LDPM is reported in Cusatis et al. (see [17,18]). In the original publication, the LDPM is defined as a meso-scale concrete model. This terminology chosen by the developer of LDPM has always been a bit controversial since the common understanding for a meso-scale concrete model is a model that simulates the mechanical properties of aggregates and cement paste and their interaction, including the formation of interface damage. The LDPM does not truly model the concrete at the mesoscopic scale. It models the concrete on the macro-scale with one set of material properties for the smeared response of aggregate, cement paste and interfaces. However, the discretization is chosen such that the main mesoscopic features like the aggregate size distribution and, hence, automatically realistic damage localization are retained, while reducing the computational cost.

The generation of the geometrical representation of the LDPM structure follows the procedure explained below. The discrete mesh is dependent on the mix design and particularly on the minimum aggregate size $d_{m}$, on the maximum aggregate size $d_{M}$ and on the Fuller coefficient, which defines the sieve curve of the aggregates. The LDPM mesh building process starts from the placement of supporting nodes (with zero radius) on the surface of the concrete domain by allowing a minimum distance of $1.1\;d_{m}$ to minimize the geometrical bias of the discretization. These nodes are placed to facilitate the internal particle placement and the application of boundary conditions. After that, the aggregates, which are approximated as spheres, are randomly placed in the concrete domain. To generate a statistically isotropic random meso-structure, the centers of particles are placed throughout the volume of the specimen one by one (from the largest to the smallest) by using a procedure introduced in the concrete literature by Bažant [24] and also used by Cusatis et al. [25]. Before the placement of any particle, a check is made for possible overlaps of this particle with previously-placed particles and with the surface nodes. A lattice mesh is created connecting all of the centers of the aggregates and surface nodes. A three-dimensional domain tessellation creates a system of polyhedral cells based on the Delaunay tetrahedralization of the generated particle centers. The polyhedral cells interact with each other through the triangular facets in common, and a lattice mesh. In LDPM, rigid body kinematics are used to describe the deformation of the lattice/particle system and the displacement jump $⟦u_{C}⟧$. At the centroid of each facet, the strain in the local normal direction is $\epsilon_{N}=n^{T}⟦u_{C}⟧/l$, while for the tangential directions they are $\epsilon_{L}=l^{T}⟦u_{C}⟧/l$ and $\epsilon_{m}=m^{T}⟦u_{C}⟧/l$. The factor *l* is the distance between the two particles; n, l and m are the unit vectors, which define the local coordinate system of any facet. The behavior of the material is defined with a vectorial constitutive law applied on the centroid of each facet. In the elastic regime, the normal stress is proportional to the normal strain through a parameter $E_{N}$ termed the normal modulus, and the shear stresses are proportional to the shear strains through $E_{T}$ where $E_{N}=E_{0}$ ($E_{0}=$ effective normal modulus) and $E_{T}$ is the product of $E_{0}$ and α (α= shear-normal coupling parameter). The three strain components are corrected by eigenstrain values, which might arise from a variety of phenomena such as, but not limited to thermal strains, shrinkage [26], expansion due to alkali-silica reaction (ASR) [20] or corrosion [27]. Visco-elasticity is treated as a standard elasticity problem by introducing additionally equivalent eigenstrains for the creep-related increase in strains. Beyond the elastic limit, the LDPM formulation considers: fracture and cohesion, compaction and pore collapse and friction. The fracturing behavior is characterized by tensile normal strains ($\sigma_{N}>0$). Effective stress ϵ and effective strain σ are defined as $\epsilon =\sqrt{\epsilon_{N}^{2}+\alpha \left(\epsilon_{M}^{2}+\epsilon_{L}^{2}\right)}$ and $\sigma =\sqrt{\sigma_{N}^{2}+\alpha \left(\sigma_{M}^{2}+\sigma_{L}^{2}\right)}$ and used to define the behavior in tension. The effective stress is incrementally elastic ($\dot{\sigma }=E_{0}\dot{\epsilon }$) until it reaches the boundary, which is defined by an exponential softening law in pure tension. The shear-tension interaction is represented through a parabolic variation of strength while the response in pure shear is assumed to be perfectly plastic. In compression ($\sigma_{N}<0$), the system response is elastic until a strain-dependent boundary, which depends on the volumetric strain, $\epsilon_{V}$, and the deviatoric strain, $\epsilon_{D}=\epsilon_{N}-\epsilon_{V}$, is reached. The volumetric strain is computed by the volume variation of the polyhedral cells. Beyond the elastic limit, pore collapse is modeled as a linear evolution of stress for increasing volumetric strain with a modulus $H_{c}$, which depends on the meso-scale compressive yield stress and other LDPM parameters. In the presence of compressive stresses ($\sigma_{N}<0$), the shear strength increases due to frictional effects. As often done in the literature, frictional phenomena can be simulated effectively through classical incremental plasticity as shown in [28].

### 2.3. Particle-Anchor Interaction

**Figure 3:**
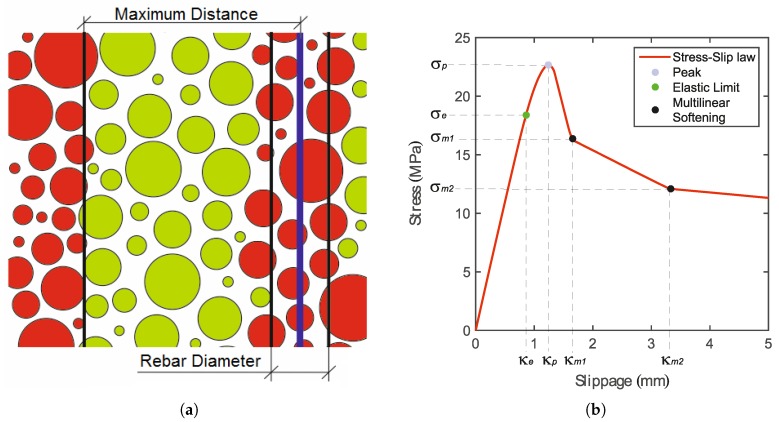
(**a**) 2D representation of threaded bar (blue), potential lattice discrete particle model (LDPM) particles connected with it (green) and non-connected particles (red); and (**b**) stress-slip law definition.

A stress-slip constitutive model [27] describes the interaction between concrete particles and the beam elements representing the anchor. These beam elements are placed inside the concrete domain without actually modeling a borehole, a convenient and computationally-effective simplification that has been shown to suffice for many practical problems associated with reinforced or prestressed concrete. The anchor elements are constrained to the surrounding LDPM particles through a virtual cylindrical surface representing the anchor surface (see Figure 3a). This surface is discretized by quadrilateral elements and has zero thickness.

As already described above, the LDPM domain is composed of a number of cells that interact through contact facets used for the definition of constitutive equations. The movement of a general point **x** inside a cell, which moves rigidly with the cell, can be expressed in terms of velocity as: $v\left(x,t\right)=v_{P}\left(t\right)\times \omega_{P}\left(t\right)\times \left(x-x_{P}\right)$, where $x_{P}$ is the position of the center of the cell, and $v_{P}\left(t\right)$ and $\omega_{P}\left(t\right)$ are the velocity and the rotation rate of the particles. The velocity of a general point $x_{A}$ can be expressed as $v\left(x_{a},t\right)=v\left(x_{A},t\right)+\omega \left(x_{A},t\right)\times \left(x_{a}-x_{A}\right)$ where $x_{A}$ is the projection of $x_{a}$ on the axes of the anchor, and $v(x_{A},t)$ and $\omega (x_{A},t)$ are the velocity and the rotation rate of $x_{A}$. The displacement is obtained from the velocity through integration in time.

Having no borehole modeled, each point on the virtual anchor surface has a companion point that belongs to an LDPM cell. The relative displacement u and velocity v of the points are defined as $u\left(x,t\right)=u_{c}\left(x,t\right)-u_{a}\left(x,t\right)$ and $v\left(x,t\right)=v_{c}\left(x,t\right)-v_{a}\left(x,t\right)$ where the subscripts *c* and *a* denote concrete and anchor points, respectively. The internal elastic energy of the constraints along the anchor axis is a bilinear positive definite symmetric operator: $a\left(u,u\right)=\frac{1}{2}\int_{S}^{}K\;u\cdot u\;ds$ where *K* is the stiffness of the concrete at the anchor interface and *S* is the entire bar length. The total work performed per unit of anchor length, added to the internal energy, must be zero. From this balance, for an arbitrary virtual displacement function, g=-ku∀x∈S is obtained. This expression can be discretized, for computational purposes, in segments with length δs. At the midpoint of every segment, a control point is placed obtaining f=Kδsu(x). This formulation can be extended to different constitutive equations such as the one used for modeling rebars subjected to corrosion [27], fiber-reinforced concrete [29,30] or the one used for bonded anchors presented in the next paragraphs.

### 2.4. Bond Law

What is macroscopically perceived as bond is the result of many mutually-interacting components and mechanisms. These are deformations in and the damage of a thin mortar layer, adhesion and interface damage between steel and mortar, as well as between mortar and concrete. In the latter case, also the surface roughness, depending on the borehole creation, the cleaning and the permeation of the mortar into the concrete may play a role. At later damage states, when the interface has failed, frictional effects further contribute to the load transfer. For the purpose of this investigation, all of the above phenomena are collectively captured by a shear stress-slip law, neglecting the effects of transversal stresses. The basic response is elastic, with stiffness $K_{a}$ in the axial direction and $K_{r}=K_{c}=p_{K}K_{a}$ in the radial and circumferential directions, where $p_{K}$ stands for the penalty coefficient. Similar to other damage models, the model assumes stiffness degradation based on the scalar damage variable ω. If isotropic damage is assumed, all stiffness moduli decrease proportionally and independently of the loading direction. The damaged stiffness is given as $K_{\left(\cdot \right)}^{d}=\left(1-\omega \right)K_{\left(\cdot \right)}$ where (·)=a,r,c. The damage evolution law is postulated in an explicit form, relating the damage variable ω to the largest previously-reached equivalent axial slippage (κ) as:

(1) $$\omega =1-\frac{\sigma (\kappa )}{\kappa K_{a}},$$

where σ(κ) is evaluated based on the user-defined stress-slip pairs; see Figure 3b. Note that stress-slip pairs have to be defined in such a way that the evaluated damage variable increases with increasing parameter κ. The pre-peak behavior is characterized by the power law as:

(2) $$\sigma \left(\kappa \right)=\sigma_{p}-\left(\sigma_{p}-\sigma_{e}\right){\left(\frac{\kappa_{p}-\kappa }{\kappa_{p}-\kappa_{e}}\right)}^{\alpha },$$

where $\kappa_{e}$ is the limit for the elastic behavior and $\kappa_{p}$ and $\sigma_{p}$ characterize the peak of the stress-slip law (Figure 3b). α is the material parameter, which governs the pre-peak hardening part of the power law. The post-peak behavior is described by multi-linear softening. The resulting increments of interface stresses are calculated as:

(3) $$\sigma_{\left(\cdot \right)}=K_{\left(\cdot \right)}^{d}s_{\left(\cdot \right)}$$

where *s* is the slippage.

## 3. Experimental Campaign

A typical bonded anchor system describes a threaded steel bar that is installed in a typically drilled hole in a concrete member. Loads are transferred through a thin adhesive mortar layer. Unfortunately, direct system tests only allow limited insights into the behavior of such systems that are typically limited to surface deformations and global response quantities. Numerical simulations provide a remedy and can represent powerful tools that give direct access to the three-dimensional distribution of stresses and strains, if the model is properly calibrated. An unbiased model calibration is a prerequisite for truly predictive investigations, and it requires a number of different tests on all involved materials, as well as for the system in order to obtain unique estimates of all of the relevant concrete and bond properties. It is essential to keep some of the experimental data for the later validation. Concrete compressive strength, tensile strength, elastic modulus and fracture energy are obtained for the three concretes at 3, 8 and 28 days. On the same days, confined and unconfined pull-out tests (the explanation of the terminology is in the related paragraphs) were performed using M12 threaded bars with a 90-mm embedment depth. The steel class of the threaded bar used was 12.9 with a yield strength of 1080 MPa, which is the strongest commercially available steel quality in order to remain in the elastic regime throughout the entire test. For all of the pull-out tests, the borehole diameter was 14 mm. Additionally, at 28 days, tests on anchors with 20 mm of unbonded length at the loaded end were performed for three different embedment depths. The concrete cone shape was recorded by photogrammetric means. Additionally, some cores were taken from the concrete slab after the pull-out test, centered at the original location of the anchor, in order to compare the simulated and actual failure mechanisms.

### 3.1. Concrete Properties

A complete characterization of concrete properties entails on top of standard tests such as cube compression and cylinder compression test (the latter is used also for the determination of the elastic modulus) also notched three-point bending tests for the determination of fracture properties and Brazilian splitting tests to obtain the indirect tensile strength. The results of the tests that were performed at a concrete age of 28 days are shown as either stress-strain or load-CMOD (crack mouth opening displacement) curves.

**Figure 4:**
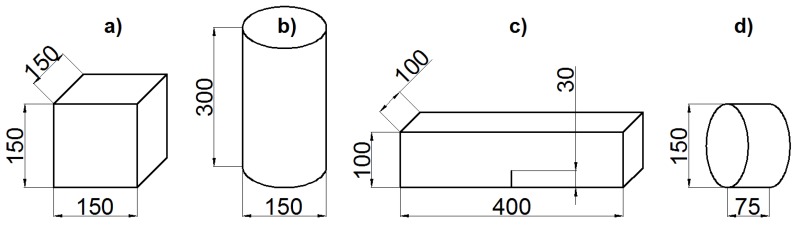
Geometry of the specimens used to determine: (**a**) compressive strength; (**b**) elastic modulus; (**c**) fracture energy; and (**d**) indirect tensile strength.

Figure 4 (dimensions in mm) shows the specimens used to determine the concrete properties. For the compression tests, three specimens were tested, while four specimens were tested for both three-point bending and Brazilian splitting.

Table 1 shows the results of the concrete characterization tests (compressive strength $f_{c}$, tensile strength $f_{t}$, elastic modulus *E* and fracture energy $G_{F}$) for the previously listed specimens. The concrete compressive strength is obtained from the cubic specimen peak-load divided by the cross-section of the specimen (see Figure 4a). The elastic modulus was obtained from the loading branch of a cylinder compression test Figure 4b) having three LVDTs placed in a 120-degree configuration with a 100-mm base length. The elastic modulus is obtained from the slope of a linear function, which was fit to the data belonging to the ascending branch of the stress-strain curve, within a range of 10–30% of the stress at peak. Figure 4c shows the dimensions of the specimens used for the three-point bending tests. The span of the tests was 300 mm. The test has been controlled by crack mouth opening (CMOD) measured by an extensometer mounted across the notch in order to ensure stability and to obtain the complete experimental post-peak curve. The indirect (splitting) tensile strength was obtained from a cylinder slice (see Figure 4d) according to [31]. All of the load-CMOD or stress-strain curves are presented in Figure 6, where also the numerical results are plotted. In the figures, the mean value is provided for peak load or stress ($F_{max}$, $\sigma_{max}$), and for the slope (*K*) of the linear part.

### 3.2. Bond Properties Determination

**Figure 5:**
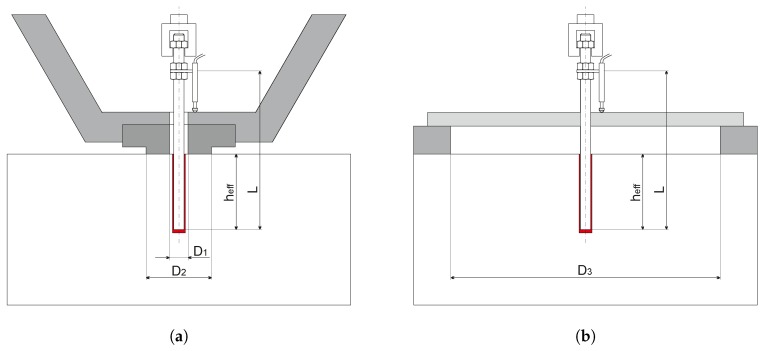
Test set-up of: (**a**) confined pull-out tests and (**b**) unconfined pull-out tests.

A thin layer of adhesive mortar is used to bond the threaded bar to the concrete. The bond behavior is determined by the material properties of the mortar, its geometry and the properties of the two interfaces between concrete and mortar and mortar and steel. The material properties of adhesive mortars include tensile, compressive and shear strength, two elastic constants and fracture properties. Reducing the mortar layer to an interface reduces the number of relevant properties to in-plane interface stiffness and strength and parameters describing its softening behavior. These properties were identified based on pull-out tests with close support, which are typically referred to as confined tests. For the confined test, a ring with an inner diameter of $D_{1}=22$ mm and an outer diameter of $D_{2}=36$ mm was used, as can be seen in Figure 5a. This configuration forces the failure to start at one of the two interfaces (concrete-adhesive mortar or adhesive mortar-steel), depending on the bond strength of the tested adhesive mortar. For the studied adhesive mortar, the bond between adhesive mortar and threaded bar is stronger than the one with the concrete. Thus, the failure starts at the concrete-adhesive mortar interface. The failure proceeds on the same interface, and at some point, it crosses the mortar layer, continuing at the mortar-steel interface. The higher the bond strength is, the longer the crack will proceed along the external interface before crossing the mortar layer. The test is controlled with two LVDTs placed consistently at a distance of $L-h_{eff}=90$ mm from the concrete surface on the loaded end. This setup leads to a more stable and controllable test and allows one to obtain a stable post peak until the load approaches zero. It is important to obtain the post peak behavior in order to properly calibrate the bond-law. Four confined pull-out tests have been performed based on which the mean maximum load, $N_{c}=$ 75.1 kN, was obtained. The embedment depth of the M12 bonded anchor is 90 mm. The mean bond strength is $\tau_{c}=22.15$ MPa, calculated under the assumption of a uniform bond stress distribution, which can be calculated by $\tau_{c}=N_{c}/\left(\pi \;h_{eff}\;d\right)$ where $N_{c}$ is the maximum load of a confined pull-out test, *d* is the nominal diameter of the threaded bar and $h_{eff}$ is the embedment depth. All values are summarized in Table 2. For the investigated adhesive mortar, the failure starts at the concrete-adhesive mortar interface. Within 2–3 mm, the crack crosses the adhesive mortar layer, reaching the adhesive mortar-steel interface. Once the crack reaches the inner interface, it travels along it, from one thread peak to the next, until the end of the anchor. Complete load-displacement curves of confined tests are shown in Figure 7a along with the related numerical results, where for both figures, the values on the *x*-axis represent the average displacement measured by the two LVDTs. In the figures, the mean value is provided for the peak load, $F_{max}$, and the mean slope, *K*, of the linear part.

### 3.3. Unconfined Pull-Out Test

Figure 5b shows the set-up of an unconfined pull-out test. The difference between a confined and an unconfined test is the distance of the support to the anchor. Guidelines state that in order to avoid any influence on the test from the supports, the width of the supports needs to be bigger than four-times the embedment depth. A ring with an internal diameter of $D_{3}=400$ mm has been used to support the slab. Considering an embedment depth of $h_{eff}=90$ mm, the boundary conditions are expected to have negligible influence on the test results. The tests are again controlled by LVDTs, which measure the relative displacement between the anchor and supporting ring through a beam that crosses the ring. For the unconfined, as well as for the confined test, the post peak response provides important insights into the system response and the failure mechanisms and, thus, is also valuable for the comparison with the simulation results. Post peak is generally important also for the simulation of anchor groups since some of the anchors in the group can reach a post peak state before others even reach the maximum load. The mean pull-out load of the unconfined test based on four samples is $N_{u}=$ 53.8 kN, as shown in Table 2. The mean bond strength is $\tau_{um}=15.87$ MPa calculated under the assumption of an equivalent uniform bond stress distribution, which can be calculated by $\tau_{um}=N_{u}/\left(\pi \;h_{eff}\;d\right)$. Complete load-displacement curves are also shown in Figure 7b along with the related numerical results for the unconfined test setup.

In Table 2, the coefficient of variation (COV) of the experimental results is shown along with the mean values. The COV of the unconfined tests is around 2% and is smaller than the one of the confined tests, which is around 5%. Typically, lower scatter is expected for the confined tests compared to the unconfined tests, which is strongly influenced by the scattering concrete properties and the concrete meso-structure.

### 3.4. Confined Pull-Out Tests of Anchors Partially Unbonded at the Loaded End

In order to extend the model validation to different embedment depths, five pull-out tests were performed in a confined configuration for each of the three different bonded lengths $b_{L}=70$, 90 and 110 mm, also using M12 threaded bar (steel class: 12.9). The setup of the test can be seen in Figure 8a. $D_{1}$ is the internal diameter of the supporting ring; $D_{b}$ is the diameter of the borehole; and $D_{t}$ is the nominal diameter of the threaded bar. The test series has been performed on anchors with an unbonded length $u_{L}=20$ mm from the concrete surface ensuring that the crack starts in the inner interface (steel-mortar). It is assumed that the unbonded length of 20 mm does not affect significantly the test results. Table 2 shows the comparison between the experimentally-determined pull-out maximum loads $N_{\left(\cdot \right)}$ and the respective bond strengths $\tau_{\left(\cdot \right)}$ where (·) represents the bonded length in mm. The latter is calculated under the assumption of an uniform bond stress distribution, which is quite consistent across different tested bonded lengths. The load-displacement curves for the unconfined tests are presented in Figure 8 along with the related numerical results.

## 4. Model Calibration

The prerequisite concerning the modeling strategy is that, to obtain an accurate bonded anchor model response, all of the parameters of the materials involved in the fastening system need to be correctly calibrated, and experimental boundary conditions need to be well reproduced. As already mentioned in the introduction, the calibration will start from the concrete material. The calibration of the bond-law (stress-slip parameters) can only be performed after the concrete calibration and validation because the model used for the bond-law calibration involves concrete (with potentially evolving damage). Once all of the material properties are calibrated, the system model will be validated on a set of unused experimental data without changing or tuning any of the parameters.

### 4.1. Calibration and Validation of Concrete Model

**Figure 6:**
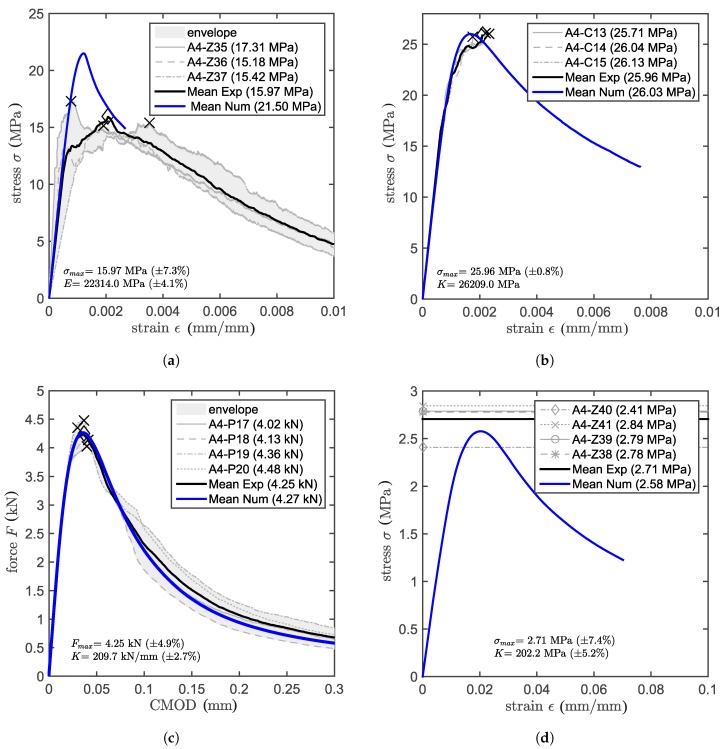
Numerical (Num) and experimental (Exp) results: (**a**) cylinder compression test; (**b**) cube compression test; (**c**) three-point bending test (**d**) Brazilian splitting test. CMOD, crack mouth opening displacement.

The LDPM needs two sets of parameters as input, one to create the discrete mesh and the other to determine the mechanical response. The discrete mesh is based on the aggregate size distribution and the mix design, which determines the total amount of aggregates to be placed (see Table 3), namely cement content (*c*), water to cement ratio (w/c) and aggregate to cement ratio (a/c) along with information about the aggregates ($d_{m}$ as minimum aggregate size and $d_{M}$ as maximum aggregate size). The maximum aggregate size and the Fuller coefficient (which is 0.5) describe the sieve curve. The minimum aggregate size represents the lower cut-off of the particle distribution, i.e., the diameter under which the aggregates are not discretely placed any more. This is an essential parameter related to the resolution of the model (ability to discretize small features) on one side, but also the number of elements influencing the computational cost on the other side. The second set of inputs is composed of the meso-scale concrete parameters. The macroscopic concrete properties available for the model calibration are the modulus of elasticity *E*, cube compressive strength $f_{c}$ and total fracture energy $G_{F}$. Since the model is formulated on the meso-scale [17], the macroscopic properties of the concrete cannot be used directly. Instead, the meso-scale properties have to be determined through inverse identification by simulating the standard material tests for fracture and compression. At the beginning of the optimization loop, two of the parameters can be already identified. The meso-scale elastic modulus is related to the macro-scale elastic modulus with a relation that is given in [17]. The meso-scale tensile strength can be approximated by the tensile strength derived experimentally from the splitting test. The optimal set of model parameters needs to concurrently provide a good approximation for the tests of elastic modulus, fracture energy and compressive strength. Figure 6a–c, shows the numerical results on top of the experimental ones for the concrete calibration. The main meso-scale parameters are: tensile strength $\sigma_{t}$, effective normal modulus $E_{0}$, tensile characteristic length $l_{t}$, ratio between tensile and shear strength $\sigma_{t}/\sigma_{s}$ and softening exponent *n*, which determines the initial slope of the softening curve. The calibrated values of these parameters are shown in Table 3. After this parameter set is determined, the concrete model has to be validated. This validation is performed with the tensile strength test. The tensile test is replicated numerically, and the result can be seen in Figure 6d. The numerical result (pure prediction) is very close to the mean experimental value and falls within the boundary of the experimental scatter. Additional validation is performed on the compressive strength obtained for cylindrical specimens. Since there are no reliable experimental results for this property, it has been approximated by the model code relation [32] from the cube compressive strength. The calculated experimental value is 22.07 MPa, and the numerical result is 21.50 MPa, confirming the capability of the model to provide reliable results also outside the calibration set.

### 4.2. Bond-Law Calibration

**Figure 7:**
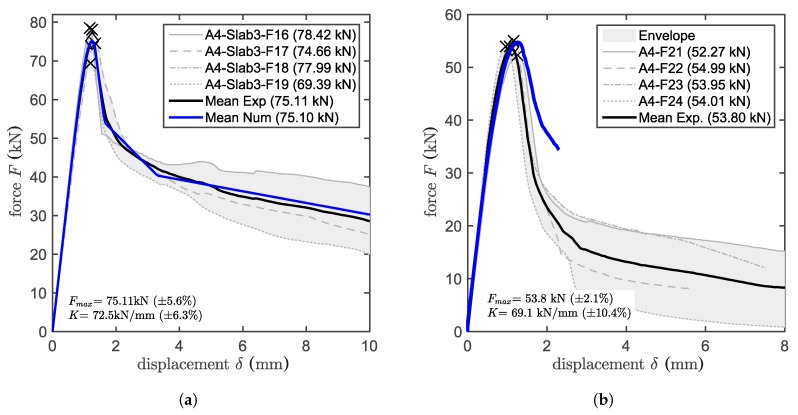
Test results of: (**a**) the confined pull-out test and (**b**) the unconfined pull-out test.

Once the concrete properties are calibrated and validated, the local bond-law parameters are calibrated. The local bond-law is applied on the integration point of each of the elements of the discretized circumferential surface defined by the anchor radius. A model for the confined pull-out test is created, utilizing the already calibrated concrete parameters, which remain unchanged. Figure 7a shows the numerical results of the mean simulated confined test on top of the mean experimental one. From the figure, it can be seen that the experimental elastic branch, the peak and the post peak have been correctly numerically reproduced. The parameter set is shown in Table 3, and the symbols are explained in Figure 3b.

The anchor model for the confined configuration simulates part of the original slab tested experimentally, which was 2.1 × 1 × 0.3 m. The simulated slab was a cube with an edge length of 0.3 m. The discrete LDPM mesh was created based on the mix design (see Table 3), the actual coarse aggregate diameter $d_{M}=$ 18 mm and the chosen minimum aggregate diameter $d_{m}$ = 4 mm. The supporting ring has been modeled just as the vertical displacement constraint on the LDPM surface nodes within a radial distance from the anchor axes of 11–18 mm. The anchor has been loaded with a velocity ramp, which grows linearly from 0–20 mm/s during the first second. After the first second, the velocity is kept constant until the end of the simulation.

## 5. Model Validation

Multiple validations were performed on the model. From the experimental campaign unconfined pull-out test results, digitized concrete cone shapes, crack patterns from cored out concrete cores (which were surrounding the position of the anchor) and pull-out tests with different embedment depth are available. Each one of these experimental results will be used for the comparison with the respective numerical results. A good model should be able to not only predict the correct peak loads, but should also reproduce accurately the deformations at peak, as well as in the softening branch of the experimental curve. Furthermore, the correct failure mechanisms should be numerically reproduced.

### 5.1. Validation on Unconfined Tests

After calibration and validation of the concrete and the calibration of the bond parameters, the quality of the model can be assessed by its ability to predict the response for pull-out tests with wide support for which either concrete cone failure or mixed failure can be expected. In the model, the wide ring support of the experiments is reproduced in order to obtain the experimentally seen combined concrete-bond failure. Figure 7b shows the numerical load-displacement curve on top of the experimental one. Four simulations were run with four different particle configurations, which as previously discussed, lead to different aggregate placement and potentially different results. The predicted peak load is 2.6% higher than the experimental one, proving the validity of the model outside its calibrated domain. Even in the post-peak domain, the predicted response follows reasonably well the experimental data, even though further improvements can be expected for more refined modeling concepts.

The anchor model for the unconfined configuration simulates also part of the original slab tested experimentally. The simulated slab was 0.7 × 0.7 × 0.3 m with the same mesh properties as used for the confined anchor model and the concrete characterization models. The boundary conditions have been defined in a similar fashion as for the confined model, but the selection of the nodes used to prescribe the vertical constraint was chosen in agreement with the dimensions of the ring used for the unconfined tests. The loading rates are consistent with those of the confined model.

### 5.2. Validation by Partially-Unbonded Pull-Out Tests

**Figure 8:**
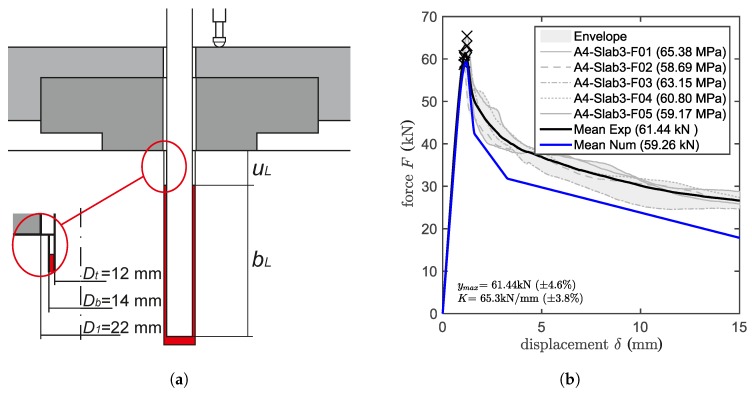

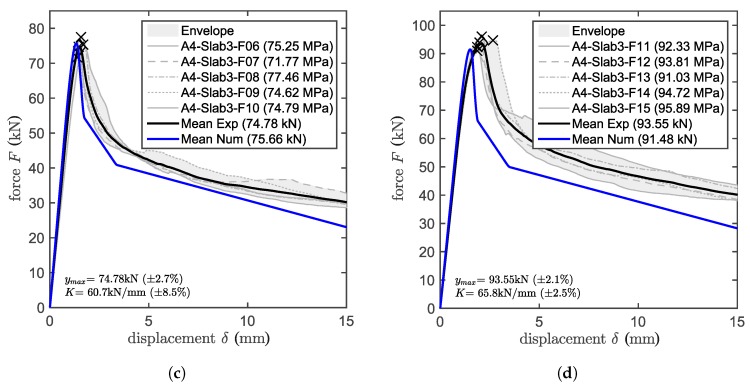
(**a**) Set-up of confined pull-out tests partially un-bonded. Experimental and numerical results for different bonded lengths: (**b**) 70 mm, (**c**) 90 mm, (**d**) 110 mm.

In order to assess the capability of the model to properly estimate the bonded anchor capacity for different embedment depths, the tests have been reproduced numerically and their results compared with the experimental ones. Simulations run for the three different embedment depths show good agreement with the experimental results. Figure 8 presents the comparison between experimental and numerical results. For $b_{L}=70$ mm, the model underestimated the experimental result by 3.5%; for $b_{L}=90$ mm, it overestimated the experimental result by 1.1%; and for $b_{L}=110$ mm, it underestimated the experimental result by 2.1%. The numerical results, being so close to the experimental ones, support the assumption that the leading interface for this type of bonded anchor is the inner one, which is the interface that should be carefully characterized for any experimental campaign. The partially un-bonded numerical models are identical to those of the confined tests (in terms of mesh, boundary and loading conditions) with the difference that the first 20 mm of the loaded end of the anchor are not bonded to the concrete member. The lengths of the anchors have been defined in the model consistently with the experimental tests (see Figure 8a).

### 5.3. Photogrammetry

**Figure 9:**
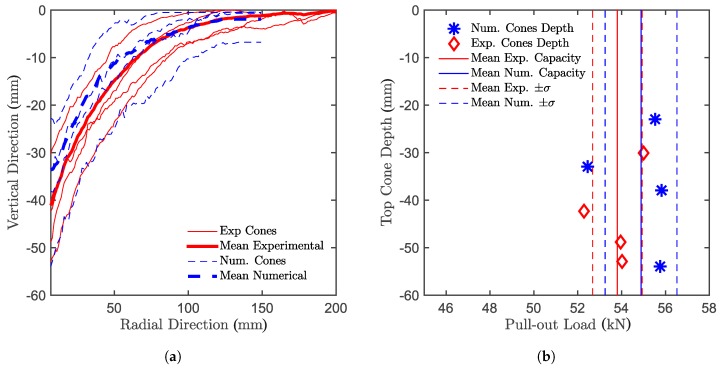
Comparison between (**a**) experimental and numerical concrete cone shape and (**b**) the relation between the top cone height and the maximum pull-out load.

A photogrammetric tool was used to obtain a virtual three-dimensional representation of the conical void left in the concrete slab after the anchors have been pulled-out. The aim is to compare the real concrete cone shape to the concrete cone shape of the numerical simulation in an attempt to validate the failure mechanism of the numerical model. A series of pictures of the conical void needs to be taken from different positions and different distances from the area of interest after a number of targets are placed around it. The positions of the targets are identified in all of the pictures, defining a common coordinate system, according to which the position and direction of the cameras for every picture taken are determined. Once the relative positions of targets and cameras are obtained, the surface of the considered object can be determined based on image correlation yielding a point cloud that represents the concrete cone surface, which can be compared with the cone shapes obtained from numerical simulations. The point cloud coordinate system is transformed from Cartesian to polar coordinates in order to be able to create a 2D diagram (see Figure 9a). On the *y*-axis, the embedment depth (vertical dimension) and on the *x*-axis the radius of the concrete cone (radial dimension) are plotted. In Figure 9a, the *x*-axis starts at 7 mm, which is the radius of the borehole, and ends at 200 mm, which is the radius of the supporting ring. The *y*-axis starts from zero, which is the concrete surface, and ends at -60 mm, which is below the deepest concrete cone (the end of the threaded bar would be at -90 mm). In the figure, the average shape of each of the four tests has been plotted along with their mean curve. On top of these, the numerical concrete cone shape is plotted showing good agreement with the experiments. Figure 9b shows the relation between the numerical and experimental concrete cone depth. From the figure, it can be seen that there is no clear relation between the depth of the concrete cone and the system capacity, both experimentally and numerically. A potential reason for this unexpected behavior will be addressed in the next paragraph.

### 5.4. Multiple Concrete Cones

**Figure 10:**
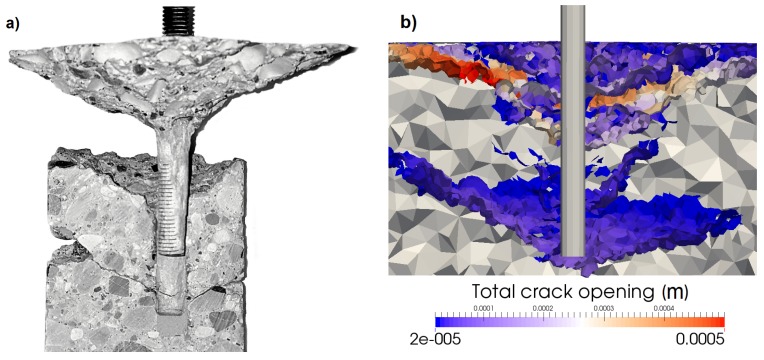
Double cone determined (**a**) experimentally and (**b**) numerically.

Depending on embedment depth and bond strength, the failure can range from complete concrete cone, to combined concrete cone-bond failure, to bond failure. For the combined failure, as can be seen in Figure 10a,b, the concrete cone breaks out, and the remaining anchor that is still bonded to the concrete will fail by bond failure. In reality, several concrete cones develop during the test. Depending on the bond strength and embedment depth, two or more concrete cones formed during the performed tests, out of which only one propagated until the surface. Figure 10a illustrates the existence of multiple cones in experiments. After the tests, a number of anchors was cored out, and the cores were sliced in half to investigate the numerically-observed failure mode. It can be seen that a second concrete cone develops emanating at the tip of the anchor and propagating for a distance longer than the radius of the cored cylinder (which is 50 mm). After the peak, one of the cones continues propagating to the concrete surface while the other ones elastically unload and close. Stronger mortar or bigger embedment depth can lead to the formation of more than two concrete cones. The numerical crack pattern is shown in Figure 10b. From the comparison between numerical results and experimental evidence, it can be seen that the secondary cone (as well as the primary, which is the one reaching the surface) is well reproduced in terms of shape and position.

## 6. Sensitivity Study

Different parameters influence a model differently; some of them can be critical, and some can be negligible. This concept applies also for experiments. A good understanding of the sensitivity between inputs and outputs allows a more accurate and efficient design of experiments or numerical campaigns. For this reason, a sensitivity study is performed on the numerical model varying the main meso-scale parameters.

During calibration, the elastic modulus and those meso-scale parameters that directly govern the concrete failure in tension and shear were inversely identified based on simulated tests that are sensitive to those parameters, i.e., cylinder compression, cube compression and notched three-point bending. The other LDPM parameters are related to mechanisms that were not investigated in this experimental campaign, such as the triaxial behavior. Consequently, suggested parameters for similar concretes were taken from the original LDPM validation paper [18], and none of them were included in the sensitivity study. The meso-scale properties considered in the sensitivity study are four calibrated damage-related parameters, namely tensile strength, tensile characteristic length, shear strength ratio and softening exponent. The description of the aforementioned parameters can be found in [17].

**Figure 11:**
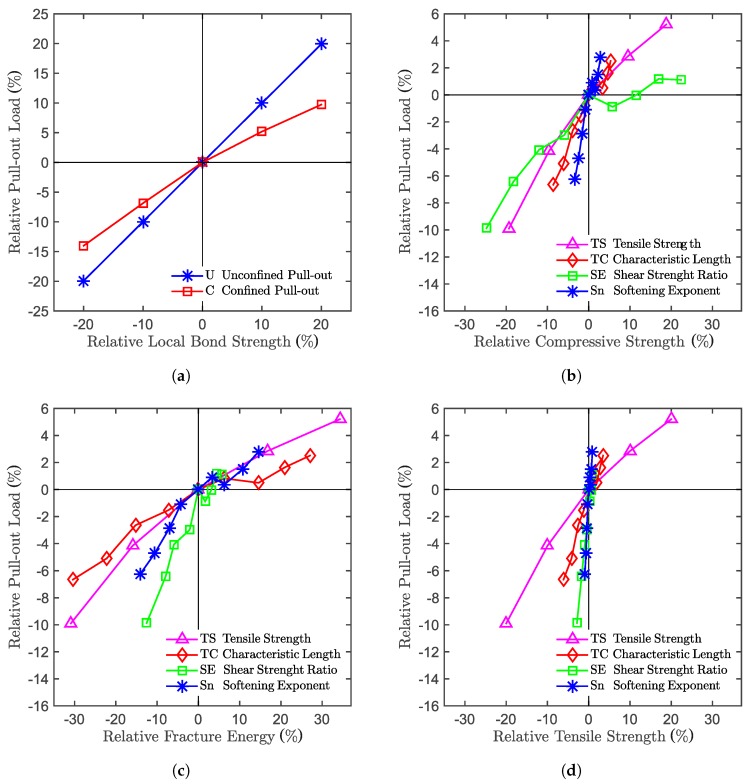
Numerical model sensitivity study. Relation between anchor capacity and: (**a**) local bond strength; (**b**) cube compressive strength; (**c**) fracture energy; (**d**) and tensile strength.

A two-step approach had to be followed for the study. In the first one, the relation between the LDPM meso-scale parameters and the macroscopic concrete properties are investigated. In the second one, the relation between the macroscopic concrete properties and the macroscopic system response are studied. Here, only the second part will be discussed, as illustrated in Figure 11. For this study, five simulations with different particle placement were run for the same parameter set in order to approach the real mean response for the given set. Figure 11a shows the relation between the local bond strength (which is a bond-law parameter) and the maximum pull-out load of the system for the confined and unconfined configurations. The local bond strength has been varied by ±20%, and in the figure, the related bonded anchor capacity can be seen to exhibit an almost linear relation. Figure 11b–d shows the relation between the bonded anchor capacity and the cube compressive strength, the total fracture energy and the tensile strength, respectively. In each of the above mentioned diagrams, the meso-scale properties that influence the macroscopic concrete properties are plotted with different colors. All of the above-mentioned meso-scale properties have been varied by ±40% in steps of 10%, except the meso-scale tensile strength, which has been varied by only two steps of 10% each. Note that the correlations between macroscopic material property and system load capacity are derived from the prescribed lower scale properties, which, e.g., do not include compressive strength. From the diagrams, it can be seen that the property that is related to the anchor capacity the most is the total fracture energy. The tensile strength, on the other hand, does not exhibit a clear relation with the system load capacity. The compressive strength shows a higher scatter compared to the fracture energy, but still, the expected trend is followed.

Generally, in Figure 11, the trend of the individual properties is not as smooth as expected. The reason is found in the limited number of simulations of different particle placements run for each parameter set. Due to the inherent variability of the discrete model, an increase in a parameter does not necessarily cause on increase in the macroscopic system response. Depending on the specific configuration of aggregates, a change in one of the parameters could trigger a different crack pattern in an early stage of the simulation, which later on could lead to a lower anchor capacity. To avoid this problem and obtain smoother and clearer trends, many more simulations with an excessive computational cost would have to be run. However, for the purpose of this sensitivity study, the trend detected is already good enough to derive significant conclusions, knowing the source of the disturbance.

## 7. Bond Stress along the Anchor

**Figure 12:**
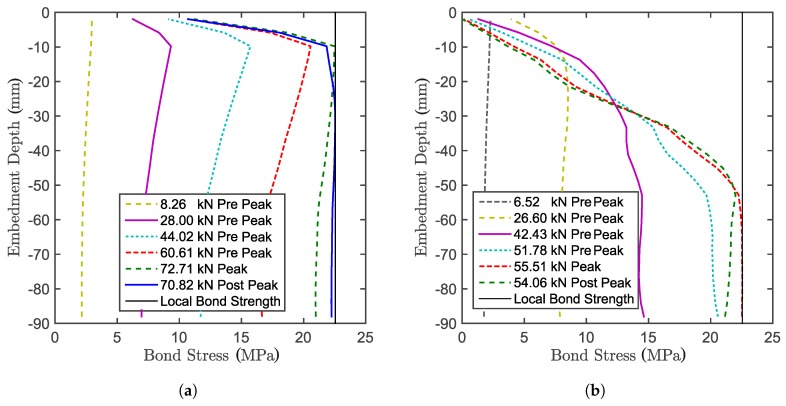
Bond stress along the anchor in numerical simulations for the (**a**) confined setup and (**b**) unconfined setup.

Figure 12 shows the difference between the bond stress along the threaded bar for confined (Figure 12a) and unconfined (Figure 12b) tests. For the confined configuration, the design hypothesis is that the stress is constant along the threaded bar. However, the only point where this statement approximately holds is at peak load. At the ultimate limit state, the stress follows the elastic solution, but has to approach zero close to the concrete surface in order to fulfil the force equilibrium. In the post-peak domain, the stress state tends to become uniform, which means that the entire interface tends to soften equally. The uniform bond is also assumed for the unconfined configuration. Unlike the confined configuration, in the unconfined case, the bond stress starts approximating a uniform state along the bar already close to the beginning of the test. Due to concrete damage however, the stress on the still bonded length progressively increases until complete bond failure is reached (which determines the system failure). This happens because the bond is stronger than the concrete, which progressively cracks, limiting the load.

**Figure 13:**
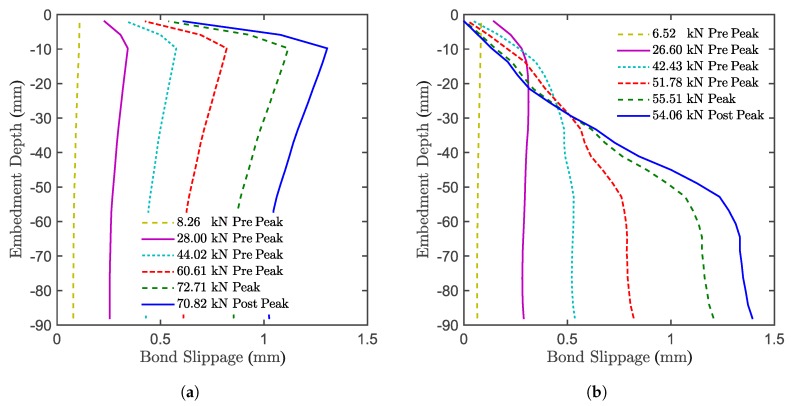
Bond slippage along the anchor in numerical simulations for the (**a**) confined setup and (**b**) unconfined setup.

Figure 13 shows the difference between the bond slip along the threaded bar for the confined (Figure 13a) and unconfined (Figure 13b) tests. For the confined tests, the slippage keeps progressing for the entire test for the entire length of the anchor, having a maximum close to the concrete surface. For the unconfined case, the concrete progressively cracks along the bar during the test. The slip between concrete and anchor reduces in the presence of concrete cracks, since locally, the stress is unloading. At the peak, where the cracks on the concrete close to the loaded end do not allow load transfer, the progression of the slippage stops. The slippage proceeds on the lower part of the anchor, where there is still load transfer through the interface.

**Figure 14:**
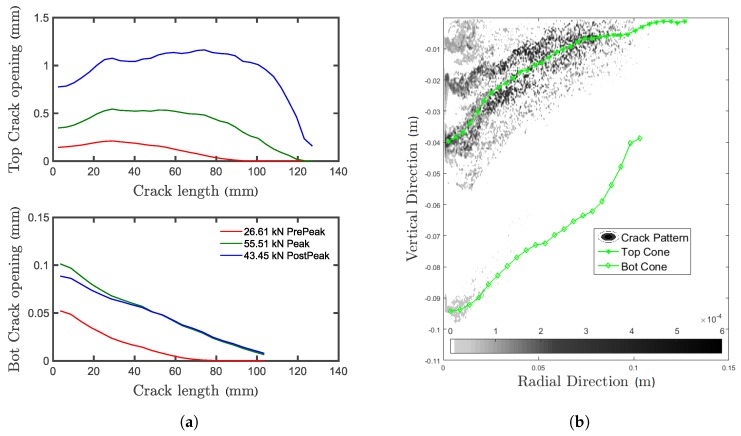
(**a**) Crack opening along the Top and Bot concrete cones of numerical simulations and (**b**) related contour plot of the crack opening.

As previously stated, more than one concrete cone is forming during the pull-out test, and all of them contribute to the system capacity. Figure 14a shows the averaged crack opening for the top concrete cone reaching the surface (labelled Top) and the secondary cone emanating at the unloaded end of the anchor (labelled Bot). From the figure, it can be seen that after the peak is reached, the Bot crack elastically closes while the top crack keeps opening, following the deformation prescribed by the testing machine. The opening of the Bot crack is one order of magnitude smaller than for the top one. Figure 14b shows the crack opening integrated in the tangential direction. It can be seen that in this case, the top cone is actually merging with another concrete cone, which starts about 20 mm above. This shows the complexity of the failure mechanisms and the benefit brought by the discrete model, which can account for the heterogeneity of the material and provides an unbiased prediction of the crack localization (independently of the mesh).

**Figure 15:**
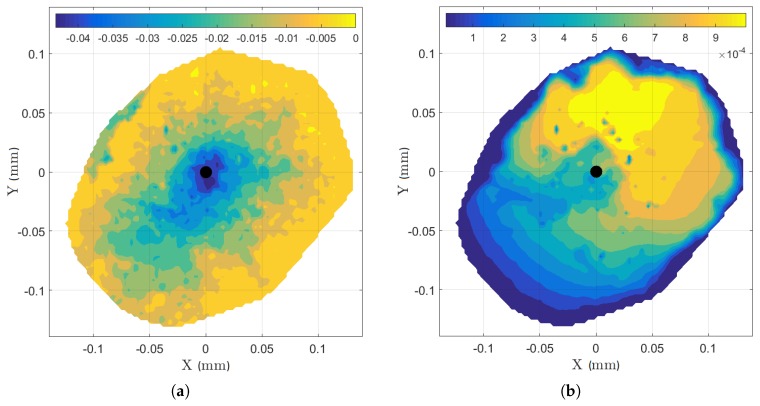
(**a**) Top view of the top concrete cone depth; and (**b**) top view of the top cone crack width (both in meters).

Concrete is a heterogeneous composite material. For normal to high strength concretes cracks mostly propagate along the aggregate interfaces and through the cement paste. The formulation of LDPM mimics this behavior and allows, together with the random placement of simulated aggregates following the actual sieve curve, to predict realistically the cone shapes. Figure 15 shows the top view of the top concrete cone depth (Figure 15a) and its crack width (Figure 15b) at peak. The coordinate system of the figures is centered on the anchor axes. As observed in the experiments, the random LDPM aggregate placement leads to a non-axisymmetric cone. Work is currently being performed to further improve the realism concerning the LDPM particle placement by mimicking the gravity effects during casting or introducing correlation between particle placement and local concrete properties (see [33,34]).

## 8. Conclusions

Tests on bonded anchors with various configurations have been performed along with a complete concrete characterization at the same concrete age. All specimens have been cast from the same batch. The tests have been used to calibrate concrete and slip-law parameters of a numerical model able to fit a designated sub-set of the available experiments. Using the same parameter set, the pull-out load, crack patterns and failure mechanisms of the unused control group have been predicted accurately. The simulated cone shapes are validated by photogrammetric measurements. Overall, the model shows a good predictive capability both in terms of anchor capacity, deformation behavior and failure mechanisms. The application of the numerical model provided novel insights into the failure mechanisms of the bonded anchor, namely the existence of multiple cones and the fact that the peak load is not correlated to the depth of the partial cone, which localizes in the case of mixed mode failure.

## Figures and Tables

**Table 1:** Experimental results of concrete tests.

$f_{c}$ (MPa)	$f_{t}$ (MPa)	*E* (GPa)	$G_{F}$ (N/m)
25.96±0.8%	2.71±7.4%	22.31±4.1%	75.0±11.2%

**Table 2:** Experimental results of tests on systems with different configurations.

$N_{c}$ (kN)	$N_{u}$ (kN)	$N_{70}$ (kN)	$N_{90}$ (kN)	$N_{110}$ (kN)
75.11±5.6%	53.80±2.1%	61.44±4.6%	74.78±2.7%	93.55±2.1%
$\tau_{c}$ **(MPa)**	$\tau_{\mathrm{um}}$ **(MPa)**	$\tau_{70}$ **(MPa)**	$\tau_{90}$ **(MPa)**	$\tau_{110}$ **(MPa)**
22.15±5.6%	15.87±2.1%	23.29±4.6%	22.05±2.7%	22.57±2.1%

**Table 3:** Calibration parameters of LDPM (top) and the stress-slip law (bottom).

Mix design	*c* (kg/m${}^{3}$)	w/c (-)	a/c (-)	$d_{m}$ (mm)	$d_{M}$ (mm)
	240	0.83	8.83	4	18
LDPM parameters	$\sigma_{t}$ (MPa)	$E_{0}$ (GPa)	$l_{t}$ (mm)	$\sigma_{t}/\sigma_{s}$ (-)	*n* (-)
	2.54	4.1	200	1.85	1
Stress-slip law parameters	$\sigma_{e}$ (MPa)	$\sigma_{p}$ (MPa)	$\sigma_{m1}$ (MPa)	$\sigma_{m2}$ (MPa)	$\sigma_{m3}$ (MPa)
	15.88	22.55	16.10	12.04	0.01
	$\kappa_{e}$ (mm)	$\kappa_{p}$ (mm)	$\kappa_{m1}$ (mm)	$\kappa_{m2}$ (mm)	$\kappa_{m3}$ (mm)
	0.35	0.73	1.22	3	30
